# Biochemical evolution of dissolved organic matter during snow metamorphism across the ablation season for a glacier on the central Tibetan Plateau

**DOI:** 10.1038/s41598-020-62851-w

**Published:** 2020-04-09

**Authors:** Lin Feng, Yanqing An, Jianzhong Xu, Xiaofei Li, Bin Jiang, Yuhong Liao

**Affiliations:** 1 State Key Laboratory of Cryospheric Science, Northwest Institute of Eco-Environment and Resources, Chinese Academy of Sciences, Lanzhou, 730000 China; 2 Aba Teachers University, Wenchuan, 623002 China; 3 State Key Laboratory of Organic Geochemistry, Guangzhou Institute of Geochemistry, Chinese Academy of Sciences, Guangzhou, 510640 China

**Keywords:** Environmental chemistry, Geochemistry

## Abstract

The metamorphism of snow (snowmelt process) has a potential influence on chemical and physical process occurring within it. This study carried out a detailed study on the variation of dissolved organic matter (DOM) in different stages of snowmelt in a typical mountain glacier located at Tibetan Plateau through collecting four different surface snow/ice categories, i.e., fresh snow, fine firn, coarse firn, and granular ice during May to October in 2015. The dissolved organic carbon (DOC) was observed by lost 44% from fresh snow to fine firn and enriched 129% from fine firn to granular ice, reflecting the dynamic variability in DOC concentration during snow metamorphism. The absorbance properties of each snow category are positively correlated with DOC concentration. The result of excitation emission matrix fluorescence with parallel factor analysis (EEM-PARAFAC) and Fourier transform ion cyclotron resonance mass spectrometry (FT-ICR MS) highlighted the domination of lipid- and protein-like compounds in glacial-derived DOM. The molecular composition of the DOM also exhibited a new N-containing molecular formula (CHON classes) that was enriched during snow metamorphism. This study suggests that snow metamorphism could induce a loss of DOM as well as enrich and modify the DOM.

## Introduction

Mountain glaciers are an important component of global hydrological cycle. Climate warming therefore have a major impact on regional water circulation through the increased flux of glacial meltwater. The increased glacial meltwater is recently suggested to impact on the balance of downstream ecosystems^1,2^ due to release of biogeochemically relevant nutrients from glaciers (e.g., carbon, nitrogen, sulfur, and phosphorus)^3–5^. The high biolability of dissolved organic matter (DOM) in mountain glacier meltwater was found in many studies^4–6^. Previous studies have found an important lipid- and protein-like DOM based upon molecular-level analyses^5,7–9^, which could contribute more than 60% of the glacial DOM^7^. The origin of DOM in glacier could be from atmospheric deposition and glacial debris, while more and more evidences suggest that endogenous source may dominate it^8^. The concentration and chemical composition of glacial DOM is observed to change seasonally, as illustrated by the variation in the DOM concentration and chemical composition in glacial meltwater river^10–13^. This seasonal variation in the concentration and chemical composition can be related to the variation in the runoff of glacial rivers, the cover rate of debris around the glacier, and the production rate of DOM in glaciers. Understanding the dynamic mechanisms of the DOM in glaciers is therefore important for estimating the evolution and flux of DOM and assessing their environmental effects.

The Tibetan Plateau (TP) contains the largest amount of mountain glaciers in middle latitudes and has exhibited a dramatic decline in recent decades due to climate change^14,15^. During the ablation season (from May to October in each year), snow metamorphism proceeds vary rapidly by the intervention of the melting process, creating various types of snow on the surface of the glacier^16,17^. The dissolved organic carbon (DOC) concentration in fresh snow differs from that in regular depth hoar evolved by fresh snow metamorphism^13^. A previous study also indicate that the process of snow-cover melt shows an positive impact on microbial diversity^18^. A recent study presented that glacial melt could promote the microbial transformations of DOM^8^. All these studies indicate that the properties of DOM are dynamically variated during melting process. Compared with polar ice sheets, mineral dust transported from around glacial debris and adjacent arid regions is abundant in the mountain glacier surface^19^, which would favourable supply key limiting nutrients and energy to microbial communities^20^. However, to date, the direct study on evolution of DOM composition during snow metamorphism in mountain glacier has not been investigated.

The glacial DOM has complex chemical nature and molecular characterization has become a primary research method to elucidate the evolution on details. Electrospray ionization (ESI) coupled with Fourier transform ion cyclotron resonance mass spectrometry (FT-ICR MS) has emerged as an effective method to determine molecular components of glacial-derived DOM^5,10,21^. The ultrahigh mass resolution and precision of this instrument makes it one of the most innovative methods used to resolve numerous individual molecular species from the complicated DOM mixture. Detailed molecular-level analysis of DOM could reflect the variations in DOM chemical components during snow melting process. In this study, we applied comprehensive analysis methods to glacial samples to (1) evaluate the chemical composition and origin of DOM within different surface snow/ice categories, i.e., fresh snow, fine firn, coarse firn, and granular ice which represent typical stages of snowmelt (Fig.  S1) and (2) explore the DOM evolution observed during snowmelt which is useful for understanding the potential biogeochemistry of glacial DOM.

## Results and discussion

### The changes of DOM content in snow/ice during snowmelt process

**Figure 1:**
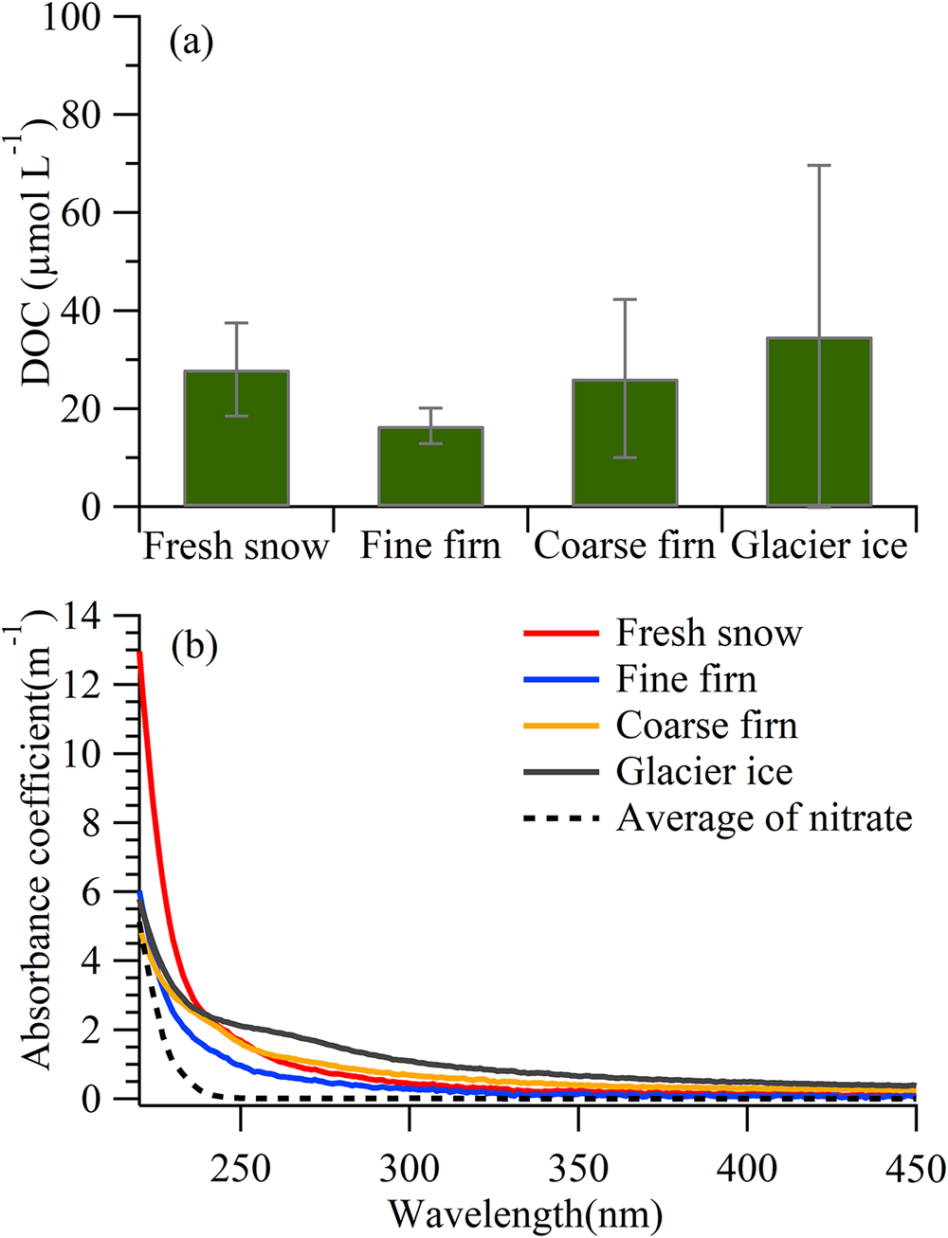
Average DOC concentration (**a**) and UV-Vis absorbance spectra of CDOM (**b**) isolated in four snow categories. The error bars indicate the standard errors (1σ).

The DOC concentrations of each snow/ice sample collected from different altitudes ranged from 5 to 189 µmol L^−1^ in each sample and there was no obvious trend related to elevation (Fig. S2), probably due to the relatively homogeneous physical characteristics of this small glacier (the length of ~2.8 km). The average DOC concentration was 28 ± 9 µmol L^−1^ for fresh snow, decreased to 16 ± 4 µmol L^−1^ for fine firn, and then increased for coarse firn (26 ± 16 µmol L^−1^) and granular ice (35 ± 35 µmol L^−1^) (one-way ANOVA, *P* < 0.05) (Fig. 1a). These variations are all statistically significant, which suggest that the DOC concentrations between different snow categories are significantly different.

The average absorbance spectrum of each category’s sample shows higher values between 220 and 450 nm, and then a sharp decrease over 240 nm (Fig. 1b). The summed values of the average absorption coefficient between 220 and 450 nm in the four categorical samples were 212 ± 90 m^−1^ for fresh snow, 116 ± 58 m^−1^ for fine firn, 170 ± 92 m^−1^ for coarse firn and 267 ± 181 m^−1^ for granular ice, which showed positive correlation (*r* = 0.98, *p* < 0.05) with their DOC concentrations (Fig. S3), indicating an increase in chromophoric DOM in the pool of glacial DOM during the melting process.

During the first stage of firnification, DOC concentration decreased from 28 ± 9 µmol L^−1^ in fresh snow to 16 ± 4 µmol L^−1^ in fine firn. The significant decrease of DOC concentrations suggested that nearly 44% of the DOC was lost downwards or released to the glacial stream in this firnification stage. A previous study also showed that the DOC in fresh snow was lower than that in regular depth hoar formed by fresh snow metamorphism^13^. Indeed, the DOC concentrations in the glacier stream at the terminus of the Laohugou No.12 (LHG glacier) were higher during early ablation season i.e. June, July and August than during the other months^11^. Lafrenière, *et al*.^22^ observed the same phenomenon, which showed that the early snowmelt samples exhibited high concentrations of hydrophobic compounds in melt water in comparison to the snow-cover. In other words, water-soluble materials were lost rapidly during the early melting period compared with other contaminants due to the percolation in the snow-cover^23^.

In further snowmelt stage, the repetitive melting and refreeze procedures lead to coarse grains^24^. Meanwhile, the DOC concentration increased significantly from 16 ± 4 µmol L^−1^ in fine firn to 35 ± 35 µmol L^−1^ in granular ice, indicating an enrichment of DOC during further snow metamorphism^19^. What are the sources of enriched DOC? During the melting season, the melted snowpack can erode the surrounding mountains and transport mineral dust to the glacial surface^19^. Then, the accumulation of mineral nutrients could contribute to the DOC concentration of snow samples. DOC in surface snow/ice could also be enriched with particles deposited from atmosphere. Meanwhile, photochemistry can increase DOC in the surface snowpack by transformation the water insoluble organic carbon into DOC by photochemical functionalization^13^. In addition, mineral dust combined with the aqueous conditions during the melting season construct a favourable environment on the glacial surface for microbes that may be *in situ* or from the aeolian biome. A previous study observed that supraglacial communities are photosynthetically active during the melting season, with production rates often exceeding respiration rates^25^; therefore, DOM could be produced and enriched (i.e., net autotrophy) during a further snowmelt period^26^. The chemical composition and molecular information of DOM below could provide details for the sources.

### The chemical composition and origin of DOM in each snow category

**Figure 2:**
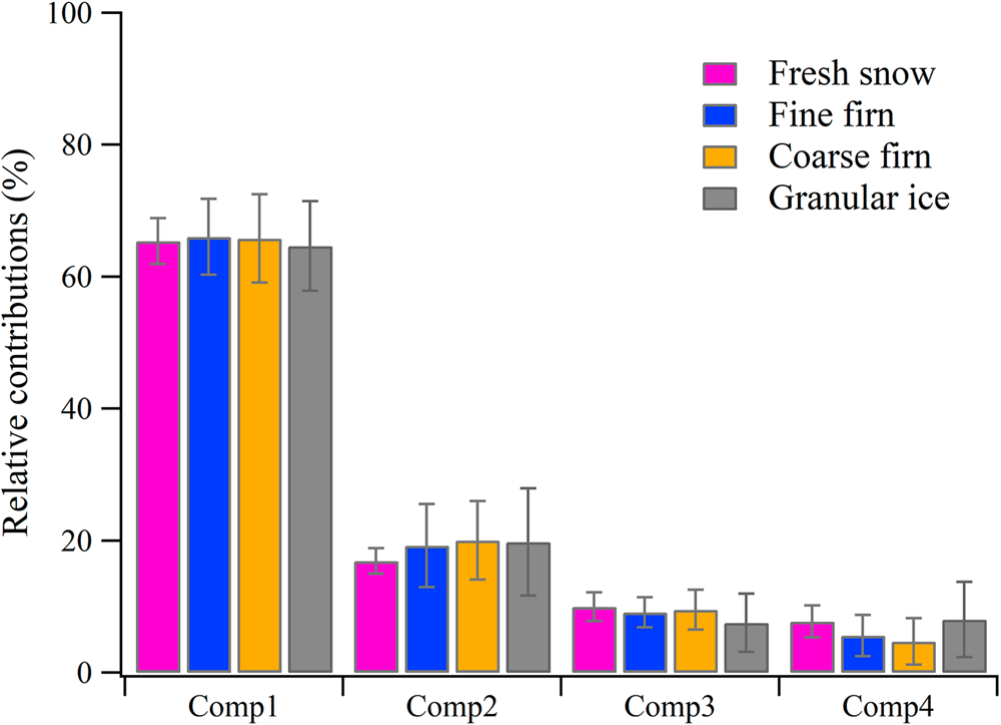
Changes of relative contribution of PARAFAC-derived DOM components in four snow categories. The error bars indicate the standard errors (1σ).

The PARAFAC model in this study identified four fluorescent components (Fig. S4) and were validated by split-half analysis (Fig. S5)^27^. The four fluorescence components included three protein-like components, i.e., C1 (tyrosine-like), C3 (trytophan-like) and C4 (trytophan-like), and one microbial humic-like component i.e., C2 (Table 1 and Fig. S4). Protein-like components were dominant in four snow categories, where the relative contribution of the three protein-like components (C1, C3 and C4) were up to larger than 80% (Fig. 2). The high containing of protein-like components in surface snow/ice might be derived from the microbe community *in situ*^28^, supported by the high values of biological index (BIX) (0.70 ± 0.08–0.80 ± 0.11) (Table 2) which indicated that biological or microbial origin mainly contributed to DOM^29,30^. This finding is consistent with other studies, which found microbially protein-like fluorescence dominating in supraglacial DOM^9,31,32^. The relative contribution of C2, which was described as low molecular weight of humic-like correlated with aliphatic carbon content and associated with autochthonous production or potential photoproduct of terrestrial DOM^33^, increased from 16.90 ± 1.91% in fresh snow to 19.79 ± 8.14% in granular ice (*P* > 0.05) likely due to photo-degradation and/or other degradation processes (Fig. 2)^34^. Note that the changes of HIX and BIX were also affected by the high solar radiation in sampling site^29^. In addition, the HIX and BIX showed no significant difference during the snow metamorphism due to the low fluorescence intensity in this study. The typical EEM spectra for fresh snow, fine firn, coarse firn, and granular ice (Fig. S6) also showed the general evolution during snow metamorphism.

**Table 1:** The peak positions for the four components identified by the PARAFAC model.

Fluorescence components	Ex/Em (nm)	Type
C1	270/315	Protein-like (tryosine-like)^32,33^
C2	<250/425	UVA Humic-like^32,33^
C3	<250 (300)/339	Protein-like (tryptophan-like)^33^
C4	290/341	Protein-like (tryptophan-like)^33^

The type for each component refers to fluorescent regions previously identified^32,33,56^. Values in brackets represent secondary peaks or shoulders.

**Table 2:** Mean values of fluorescence index (FI), humification index (HIX), and biological index (BIX) for DOM extracted from different snow categories (mean ± SD).

Snow category	FI	HIX	BIX
Fresh snow	1.69 ± 0.17	0.27 ± 0.04	0.70 ± 0.08
Fine firn	1.42 ± 0.25	0.31 ± 0.12	0.80 ± 0.11
Coarse firn	1.43 ± 0.21	0.30 ± 0.16	0.75 ± 0.13
Granular ice	1.48 ± 0.17	0.36 ± 0.24	0.75 ± 0.19

**Figure 3:**
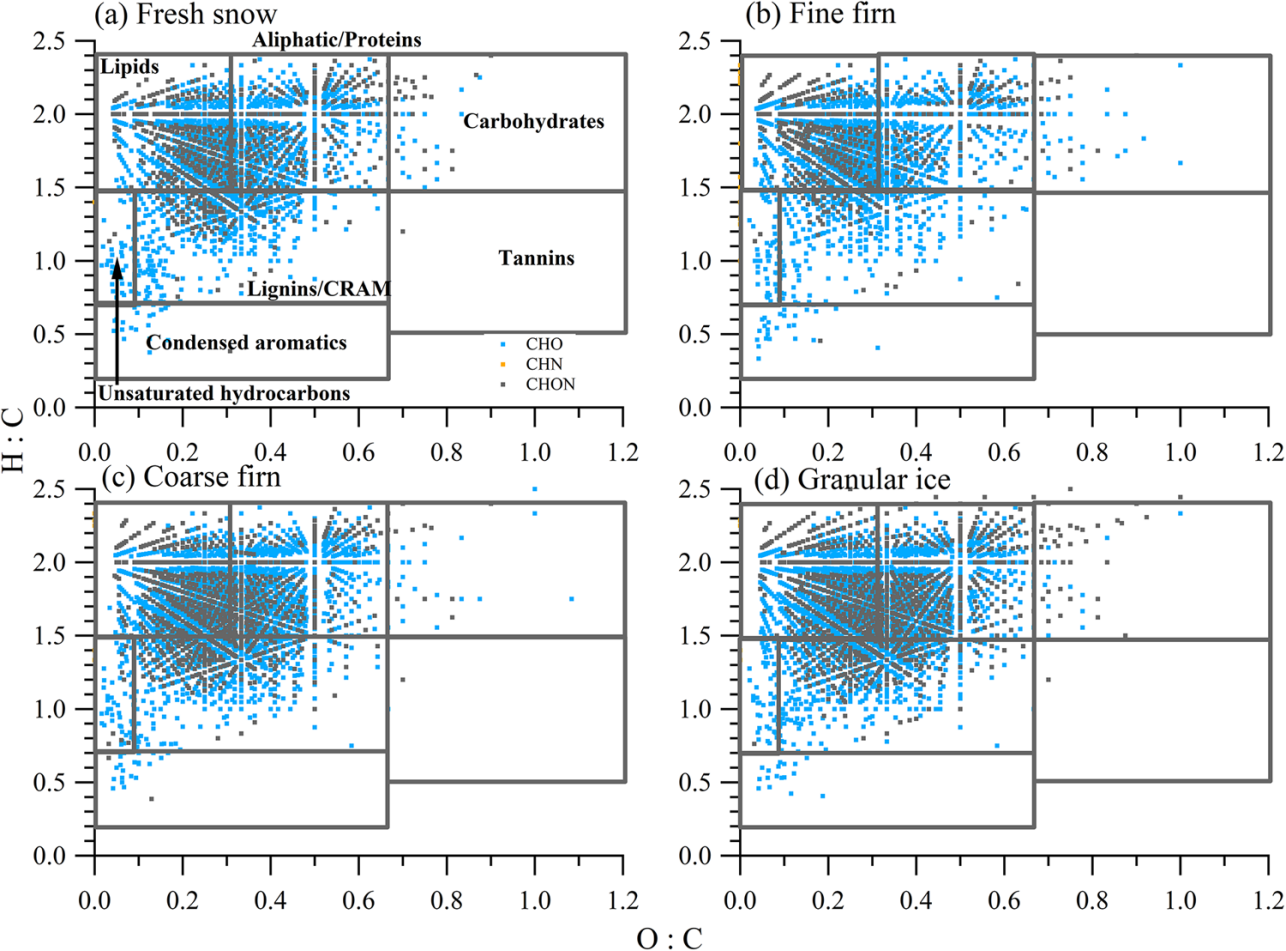
Van Krevelen diagram where blue data points indicate CHO classes, gray indicates CHON classes, and orange indicates CHN classes in four snow categories. Areas assigned as different biochemical classes of identified DOM formulas.

The van Krevelen (VK) diagram, a graphical analysis complementary to plot the elemental ratios of oxygen to carbon (O/C) and hydrogen to carbon (H/C) of complex DOM mixtures, which showed a high molecular diversity of DOM compositions in four snow categories (Fig. 3). Note that the formula in a particular category based on only the H:C and O:C ratio in the VK diagram maybe include different isomers. Three biomolecular classes (Fig. 3, Table 3), including lipids (29.29% – 41.88%), aliphatic/proteins (33.25% – 40.33%), and lignin/ carboxyl-rich alicyclic molecules (CRAM) (19.32% – 26.98%), dominated the assigned molecular compounds. The presence of lignin/CRAM content in the DKMD glacier is similar to the East Antarctic ice sheet^21^, both of which potentially originate from terrestrial sources or biomolecules that have similar characteristic with sterols and hopanoids^35^. Small contribution (0.4%−1%) of condensed aromatic compounds (Table 3), with low O:C ratios (≤0.2), and a highly modified aromaticity index (AI_mod_) (≥0.67) were also observed in the DOM extracted from the four snow categories. The condensed aromatic DOM in snow on glacier could derived from wildfires, charred soil residues^36^ or ambient particles. In general, the distribution of DOM in the van Krevelen diagrams for the four categories was dominated by lipids and aliphatic/protein-like material, which could be originated from *in situ* microbial compounds^37^.

**Table 3:** The average molecular weight, the number and the percentage (%) of molecular formulas in each biochemical compound assigned by FT-ICR-MS for DOM in different snow categories.

Snow category	Average molecular weight	Lipids (%)	Aliphatic/proteins (%)	Carbohydrates (%)	Unsaturated hydrocarbons (%)	Lignin/CRAM and tannins (%)	Condensed aromatics (%)
Fresh snow	391	921 (31.29%)	1147 (38.97%)	44 (1.50%)	52 (1.77%)	760 (25.83%)	19 (0.65%)
Fine firn	376	1034 (41.88%)	821 (33.25%)	41 (1.66%)	70 (2.84%)	477 (19.32%)	26 (1.05%)
Coarse firn	392	1116 (31.87%)	1371 (39.15%)	29 (0.83%)	78 (2.23%)	890 (25.41%)	18 (0.51%)
Granular ice	396	1147 (29.16%)	1586 (40.33%)	55 (1.40%)	67 (1.70%)	1061 (26.98%)	17 (0.43%)

A mixture of microbially and terrestrially derived DOM was observed not only in aged snow samples (firn and ice) but also in fresh snow. Previous studies observed these mixed chemical characteristics in ambient aerosols^38^ and fog water^39^ in other remote areas. During summer, the primary biological aerosol particles, such as archaea, fungi, pollen, algae and virus, would become diversity^40^ under the suitable environment, which would be relevant to the formation of clouds and precipitation^41^. Therefore, biological aerosols could be a potential source for the microbially related DOM compounds in fresh snow.

### The biochemical evolution of DOM composition during the snow melting process

The absorption coefficient observed between 240 and 450 nm in granular ice was higher than that in other category samples, suggesting a high concentration of chromophores such as aromatic amino acid in granular ice than in other categories^42–45^. The average SUVA_254_ values obtained for each category’s sample were 1.96 ± 0.40 L mg C^−1^ m^−1^ for fresh snow, 1.85 ± 0.49 L mg C^−1^ m^−1^ for fine firn, 2.39 ± 1.63 L mg C^−1^ m^−1^ for coarse firn, and 2.84 ± 1.76 L mg C^−1^ m^−1^for granular ice, which implied that DOM with lower aromaticity in snow/ice samples compared with other DOM pool (3.2–5.3 for DOM in typical river samples)^46^. The *S*_*R*_ values of DOM in each category (1.56 ± 0.05 for fresh snow, 1.76 ± 0.68 for fine firn, 1.6 ± 0.04 for coarse firn, and 1.57 ± 0.03 for granular ice) exceeded 1.0, indicating endogenous sources^47^. Moreover, decreased *S*_*R*_ values across fine firn to granular ice suggested a growth of molecular mass of the DOM which was verified by the result of FT-ICR MS (Table 3).

**Figure 4:**
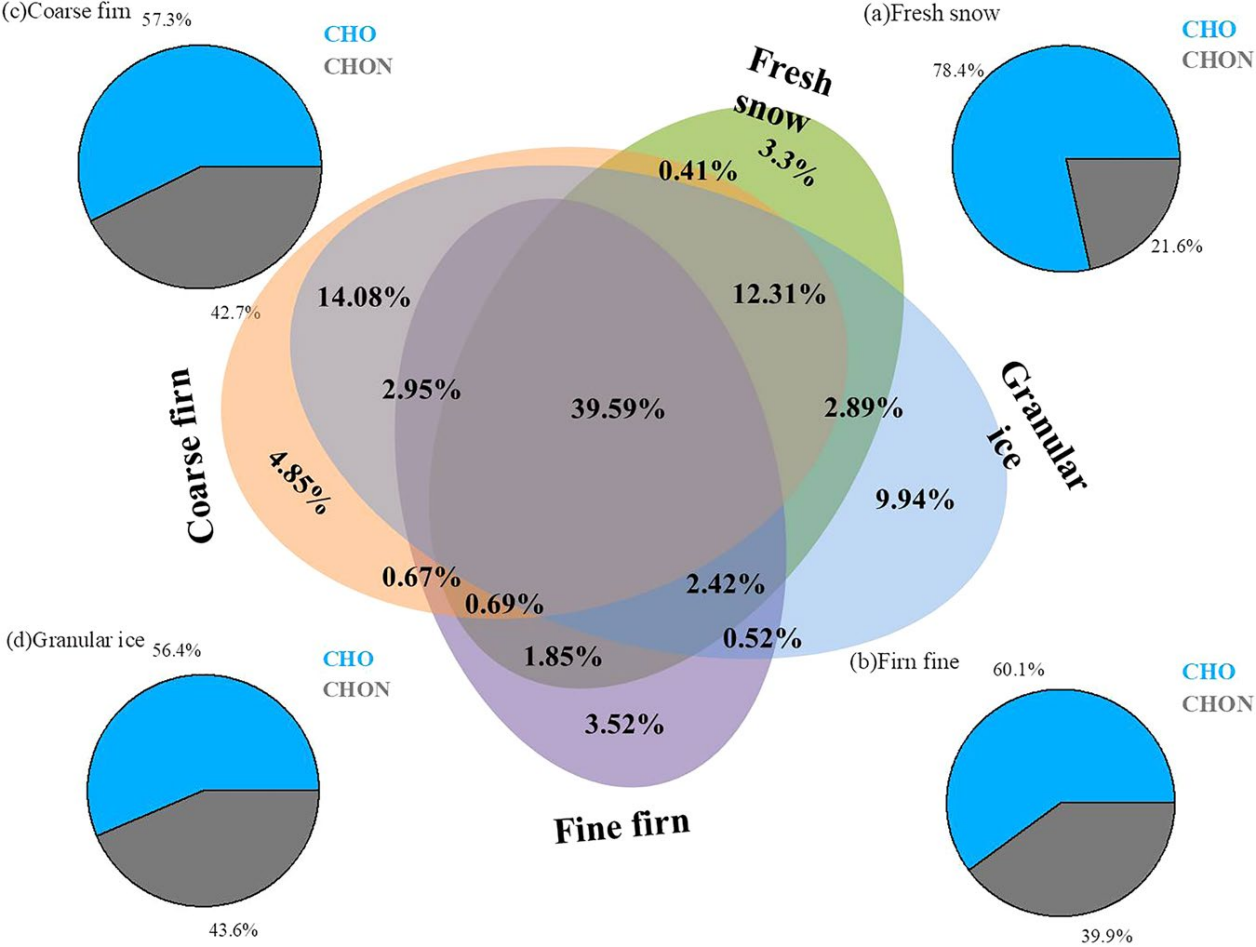
Comparisons of the formulas in the different DOM samples are illustrated by Venn diagrams that evaluate the formulas that are unique and common to four snow categories. The relative contributions of the CHO and CHON molecular classes are shown in unique components in the four pie charts.

**Figure 5:**
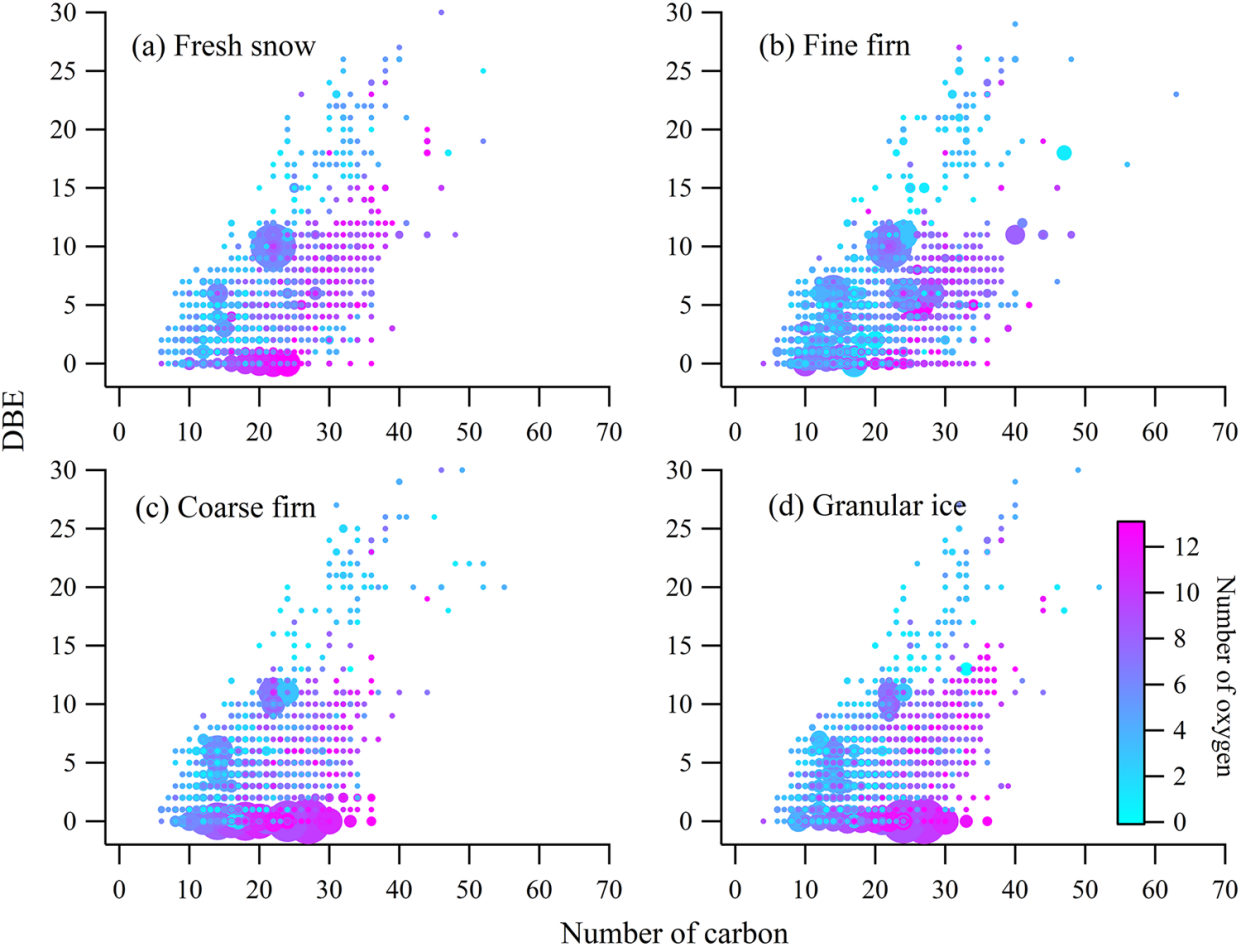
Iso-abundance diagrams of DBE versus carbon numbers for DOM molecules in four snow categories. The number of oxygen atom shown at the colour bar.

The relative contributions of fluorescence components were also changed with the snow metamorphism (Fig. 2). The contribution of tyrosine-like components (C1) decreased from 66.05 ± 5.74% in fine firn to 64.64 ± 6.79% in granular ice (*P* > 0.05) while trytophan-like (C4) increased from 5.58 ± 3.12% in fine firn to 8.05 ± 5.70% in granular ice (*P* < 0.05). This suggests that the protein-like fluorescence transformed dynamically in supraglacial environments. However, the value of relative mass content of carbon (C_m_) in each average formula are generally identical in this study (Table S3) which might be due to the domination of low average molecular weight of DOM (Table 3) and the highly similarity of DOM composition in four snow categories (Fig. 4). In addition, the high abundance of the molecules gradually transitioned from high double-bond equivalents containing (DBE-containing) to low DBE-containing across fine firn to granular ice. In this procedure, the oxygen content increased (Fig. 5) as well as the ratio of O/C increased from 0.29 in fine firn to 0.33 in granular ice, suggesting there exists degradation processes during snowmelt^48^, which implies variation in molecular structure during further snowmelt process.

The changes of DOM composition during the snowmelt were further analysed according to the distinctive and common molecular formulas in four snow categories by Venn plots (Fig. 4). A fraction of 39.59% of the total assigned molecules overlapped, suggesting that the DOM in four snow categories had a highly similarity. Regardless, granular ice shows the most distinctive molecular compounds (9.94%), followed by coarse firn (4.85%), fine firn (3.52%) and fresh snow (3.30%). In addition, the contributions of CHON molecules in unique molecular components showed an increasing trend of 21.56% in fresh snow, 39.87% in fine firn, 42.67% in coarse firn, and 43.60% in granular ice, which suggested that the snow metamorphism could alter the component of DOM. The increased contributions of trytophan-like (C4) and microbial humic-like (C2) and new CHON compounds in granular ice (Figs. 2 and 4) suggested an enrichment of microbially derived DOM in granular ice. A previous study demonstrated diverse assemblages, including bacteria, eukarya and archaea, in snowpack, which could produce diverse enzymes, i.e., protease, lipase, cellulase, β-galactosidase, amylase, and lignin-modifying enzymes^49^ and decompose and transform DOM and then release new microbial sources of DOM through microbial metabolism^8,37^. As a consequence, the biochemical evolution for DOM was observed during the snow melting process in the ablation season on the surface of the glacier which presented in a schematic (Fig. S7).

### Summary

We employed UV-Vis and fluorescent spectroscopy alongside ESI-FT-ICR MS methodological approaches on snow/ice samples to evaluate the composition, origin and variation of DOM during the whole ablation season in the DKMD glacier. The variations in DOC concentration indicated that the firnification from fresh snow to fine firn in glacier caused remarkable loss of DOC (~44%), after that the DOC was remarkably enriched (~129%) in further metamorphism from fine firn to granular ice mainly via autochthonous sources. The light absorbance of DOM accordingly followed the trends of the concentration of DOC. The EEM data coupled with PARAFAC modelling proved that the protein-like compounds dominated the fluorescent DOM. The results of FT-ICR MS revealed that the chemical composition of the identified DOM formulas primarily contained microbial-derived lipids and aliphatic/proteins compounds (amount to 69.5–75.1%) and had a lower content of terrestrial-derived lignin/CRAM compounds (19–27%). Therefore, we concluded that the enriched DOM during snowmelt was mainly from autochthonous microbial communities. In addition, the contributions of CHON molecules in unique molecular components showed an increasing trend during the snow metamorphism, suggesting that the snowmelt process could continually alter DOM composition within it. In summary, DOM undergoes biochemical evolution during snow metamorphism on the glacial surface during the ablation season.

## Methods

### Sites and sampling

**Figure 6:**
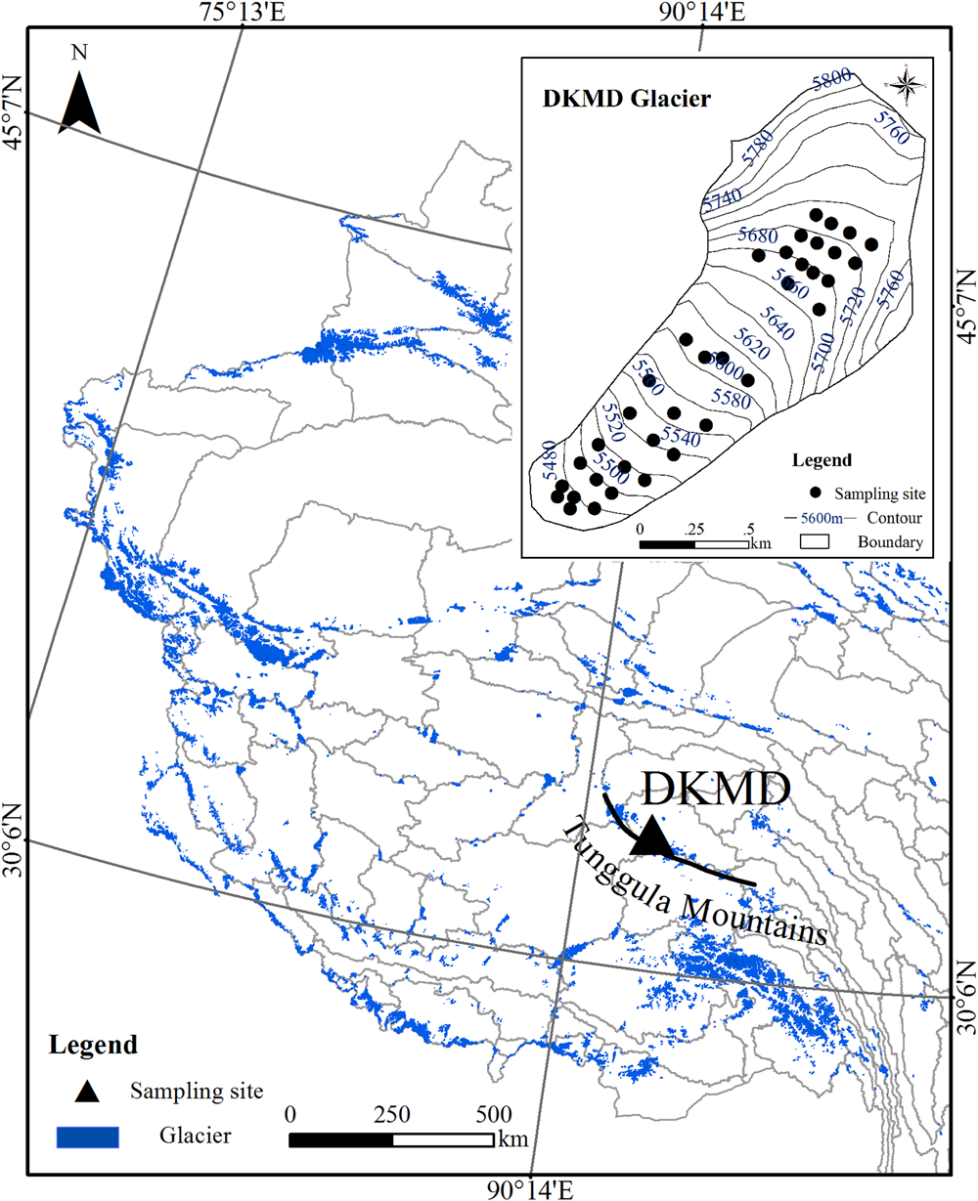
Map of sampling location with the black triangle representing the Dongkemadi (DKMD) glacier field site. The black points in small image indicate the sampling site of surface of snow/ice. The figure is generated in ArcMap (version: 10.3, https://desktop.arcgis.com/en/arcmap/) using the Second Glacier Inventory Dataset of China (Version 1.0) (http://westdc.westgis.ac.cn/data/f92a4346-a33f-497d-9470-2b357ccb4246).

The Dongkemadi (DKMD) glacier (33°05´N, 92°04´E, 5380–5926 m a.s.l.) is situated at the northern of Tanggula Pass of the central TP (Fig. 6). The glacier surface area was ~1.8 km^2^, with a length of ~2.8 km and an average width of 0.5–0.6 km in 1995. Influenced by global warming, serious ablation has happened in DKMD glacier at a mass loss rate of approximately 300 mm w.e annually during last 20 years^15,50^. The equilibrium-line altitude for the DKMD glacier has varied from 5450 m a.s.l. to 5850 m a.s.l. during 1989–2002. Therefore, most of the glacier could be below the equilibrium line altitude (ELA) during the summer, as was the case during our sampling period. Surface glacier snow/ice samples (to a depth of ~10 cm) were collected from different altitudes at ~50 m intervals during the entire ablation season (from May 2015 to October 2015) using a stainless steel scoop pre-combusted at 600 °C for 8 h. Along the horizontal glacier transect, three parallel samples on average were collected at each altitude (Fig. 6) once a month. About 36 samples were collected in every sampling month. All samples were melted within the refrigerator after collection (within one day) and passed through 0.45-µm quartz fibre filters (pre-combusted at 600 °C for 8 h) at field stations before being transported to the laboratory. All filtered samples were filled into 50 ml Falcon tubes and frozen in darkness before transportation, during which cooler filled with blue ice was used to freeze the samples. In view of the physical properties of snow/ice during the sampling, all samples were classified into four categories. The different four categories included fresh snow, fine firn, coarse firn, and granular ice (Fig. S1), representing typical stages of snow melting^51^. Note that we use these phrases used for classic snow metamorphism in the accumulation zone of glaciers to describe snow evolution in the ablation zone of glaciers.

### DOC concentration and absorbance analyses

The determination of DOC concentrations in each sample were made by Vario EL CN analyzer (Elementar, Hanau, Germany) referred as previous study^42^. The routine minimum detection limit for DOC was ∼4.16 µmol C L^−1^, assessed by the standard deviation of several ultrapure water samples. The standard errors are typically better than 5%. Procedural blanks were prepared using Milli-Q water in 50 mL Falcon tubes at the beginning of filtration and evaluated potential contamination that could occur during sampling preparation and filtration. The mean concentration of procedure blanks was 5.9 ± 1.2 µmol L^−1^.

The UV-Vis absorbance scans were measured at a range of 200–900 nm in 1 cm quartz cuvettes by a Shimadzu UV-2410PC UV-visible spectrophotometer. The detailed calculation of absorbance coefficients, absorbance coefficient of nitrate, and some optical parameters, including spectral slope (*S*), slope ratio (*S*_*R*_), and average specific UV absorbance at 254 nm (SUVA_254_), are all shown in the Supplementary Information. The nitrate contributed little to the whole absorption coefficient in every snow categories with the average value below 10%. The average absorption spectrum of nitrate was presented (Fig. 1b).

### Three-dimensional fluorescence measurements

Fluorescence spectra of all samples were measured in 1 cm quartz fluorescence cell by Hitachi F-7000 fluorescence spectrometer referring to previous study^52^. Excitation wavelengths (Ex) were scanned at a range of 230 and 450 nm in 5 nm step, and emission wavelengths (Em) was determined between 300 and 550 nm in 2 nm step. The slit widths of excitation and emission monochromators were adjusted to 5 nm with the scan speed at 2400 nm min^−1^. The detailed descriptions for the PARAFAC modeling and the calculation of the fluorescence index (FI), BIX, and HIX were given in supplementary information.

### ESI-FT-ICR MS analysis

After measurements, all snow/ice were grouped into four categories i.e. fresh snow, fine firn, coarse firn and granular ice. Then all samples were processed solid-phase extraction with 500 mg Bond Elut PPL columns (Agilent Technologies) to concentrate the DOM and desalt for FT-ICR-MS analysis^53^. The extraction efficiency of PPL-based solid-phase extraction (SPE) across all type of samples was larger than 60% based on previous study^54^. PPL-based SPE has strong adsorption capacity for both strong polarity and weak polarity DOM and covers a large polarity range of DOM^54^. Therefore, the concentration would decrease from bulk DOM to SPE-DOM, yet there was no significant difference in composition between them. All PPL cartridges were pre-cleaned and conditioned by 15 mL of LC-MS grade methanol, then successively washed by 10 mL of ultrapure water, 5 mL of methanol, and 15 mL of acidified ultrapure water (pH = 2, adjusted using LC-MS grade HCl).

All samples were adjusted to pH 2, then eluted and extracted through the cartridges at a flow rate of approximately 1 mL min^−1^. After that, columns were rigorously washed with 15 mL of acidified MilliQ water, and eluted by ~10 mL of methanol to pre-combustion (450 °C, 6 hours) vials which were finally dried by ultra-high purity nitrogen to ~1 mL and kept in a refrigerator (4 °C). Unfortunately, we only had one sample for each category due to the limited sample volume for FT-ICR MS measurement. Based on the same procedure, the procedure blank was got to check whether the samples were contaminated during the sample treatment procedure.

The extracted DOM were analysed with a 9.4 T FT-ICR mass spectrometer (Solarix, Bruker, Thermo Germany) equipped with an electrospray ionization (ESI) source. The extracted DOM were injected into the ESI source through a syringe pump at 2 μL min^−1^ to generate both negatively and positively charged molecular ions. The spectra were acquired with the m/z range of 150–800, and 100 transients were summed for each mass spectrum. The mass–resolving power at m/z 400 was greater than 400,000. Mass spectra of blanks were excluded from the peak list of samples prior to formula assignment. The molecular formula calculations was performed with a customized software developed based on the criteria in Koch, *et al*.^55^. Briefly, the formula assignment was focus on peaks according to the signal-to-noise ratio of ≥10, and mass error ≤1.5 μg g^−1^. The upper criteria about the number of atoms in each molecular formula were adjusted to thirty ^12^C, sixty ^1^H, twenty ^16^O, three ^14^N, one ^32^S, one ^32^S, one ^13^C, one ^18^O and one ^34^S. The detailed descriptions for the molecular formula assignment, the calculation of the double bond equivalence (DBE) and the identification for the biomolecular class of DOM molecules are all shown in the Supplementary Information.

## Supplementary information

Supplementary information.

## Data Availability

Correspondence and requests for relevant data should be emailed to J.Z.X. (email: jzxu@lzb.ac.cn).
