# Supplementary material for: Biochemical evolution of dissolved organic matter during snow metamorphism across the ablation season for a glacier on the central Tibetan Plateau
Source: Sci Rep. 2020 Apr 9;10:6123. doi: 10.1038/s41598-020-62851-w (PMC7145860; doi:10.1038/s41598-020-62851-w)
Supplement: Supplementary file 1 — Supplementary information. [file 41598_2020_62851_MOESM1_ESM.pdf]

Supplementary information for:

Biochemical evolution of dissolved organic matter during snow metamorphism across the ablation season for a glacier on the central Tibetan Plateau

Lin Feng[1,2], YanqingAn[1], JianzhongXu[1*], Xiaofei Li[1], Bin Jiang[3], Yuhong Liao[3]

[1]State Key Laboratory of Cryospheric Science, Northwest Institute of Eco-Environment and Resources, Chinese Academy of Sciences, Lanzhou 730000, China

[2]Aba Teachers University, Wenchuan 623002, China

[3]State Key Laboratory of Organic Geochemistry, Guangzhou Institute of Geochemistry,

Chinese Academy of Sciences, Guangzhou 510640, China

*Correspondence to: jzxu@lzb.ac.cn

1. Calculations

1.1 Bulk optical properties

Absorbance spectra were baseline-corrected by subtracting the mean absorbance for the spectral range from 690 nm to 700 nm. Optical density values (i.e., absorbance values) were converted to absorption coefficients by fitting the equation:

$$a(\lambda) = 2.303 \cdot \mathrm{A}(\lambda)/\mathrm{L} \quad (1)$$

where $\mathrm{A}(\lambda)$ is the measured absorbance for a wavelength λ, L is the pathlength of the optical cell (here, L = 0.01 m), and 2.303 is the common-to-natural logarithm conversion factor.

The DOM spectral slope (S) was calculated using a nonlinear fit of an exponential function to the absorption spectrum in the ranges of 275–295 and 350–400 nm using the following equation:

$$a(\lambda) = a(\lambda_0)e^{S(\lambda_0-\lambda)} + \mathrm{k} \quad (2)$$

Where $a(\lambda)$ is the absorption coefficient of chromophoric dissolved drganic matter (CDOM) at a specified wavelength, (λ_0) is the reference wavelength and S is the slope fitting parameter. The slope ratio (S_R) was calculated as the ratio of $S_{275–295}$ to $S_{350–400}$ as described by Helms, et al. [1]).

Note that the sample absorption coefficient $a(\lambda)$ at a given wavelength represents the sum of the absorption coefficient contributions from all chromophores, including DOM and nitrate. To eliminate the interference of nitrate on the absorption properties of DOM, we estimated the absorption coefficient of the optical absorption of nitrate.

The absorption coefficient of nitrate at each wavelength, $a(\mathrm{NO_3^-},\lambda)$ was determined for each snow sample using the following equation:

$$a(\mathrm{NO_3^-},\lambda) = \varepsilon(\mathrm{NO_3^-},\lambda) \cdot [\mathrm{NO_3^-}] \quad (3)$$

Where $\varepsilon(\mathrm{NO_3^-},\lambda)$ is the molar absorptivity of $\mathrm{NO_3^-}$ at λ and $[\mathrm{NO_3^-}]$ is the molar concentration of $\mathrm{NO_3^-}$ in the melted snow sample. We adopted the $\varepsilon(\mathrm{NO_3^-},\lambda)$ values (220-350 nm) measured in the laboratory from other study [2], and the values of $[\mathrm{NO_3^-}]$ used here were measured using ion chromatography in our laboratory.

The average specific UV absorbance at 254 nm ($SUVA_{254}$), which is an indicator of the aromaticity and chemical reactivity of DOM, was calculated by dividing the average absorptivity at λ = 254 nm by the average concentration of DOC, in units of L mg $C^{-1}m^{-1}$ [3].

A series of optical parameters, including the fluorescence index (FI), biological index (BIX), and humification index (HIX), were obtained from the EEMs. The FI is calculated as the ratio of the emission intensity at 470 nm to that at 520 nm, both of which are obtained from excitation at 370 nm [4]. A FI value of 1.4 or less indicates that the DOM is of terrestrial origin; a FI value of 1.9 or higher indicates microbially derived DOM [5]. The BIX is calculated as the ratio of the emission intensities at 380 and 430 nm, both of which are obtained from excitation at 310 nm [6]. The values of BIX falling in the range of 0.8−1.0 are characteristic of freshly produced DOM of biological or microbial origin, whereas values below ~0.6 indicate that little autochthonous organic matter is present [5]. A modified HIX is calculated using the area under an emission spectrum (acquired using excitation at 254 nm) between 435

and 480 nm and dividing it by the area under the spectrum between 300 and 345 nm plus the area under the spectrum between 435 and 480 nm; these values were used to compare the humification levels of different DOM samples [5].

1.2 PARAFAC modeling

The Raman scatter of water was calibrated by subtracting the daily excitation emission matrix (EEM) fluorescence values of the Milli-Q water blanks [7]. The Rayleigh scatter peaks were addressed by the EEMscat MATLAB toolbox (version 3) using an interpolation algorithm [8]. The UV absorbance at 254 nm was below 0.05 in all snow/ice samples [9], which made the inner-filter correction unnecessary. To remove instrument-dependent intensity effects, we present our results on a unified scale of Raman units following the method of Lawaetz and Stedmon [10]).

Parallel factor analysis (PARAFAC) statistically decomposes complex mixtures of DOM fluorophores into individual components without making any assumptions about their spectral shapes or their number. The combination of EEMs and PARAFAC has been a useful method applied to characterize DOM in aquatic environments and in glacier samples [11-14]. Each component identified using PARAFAC has a unique excitation and emission spectrum, and each component may comprise a single fluorophore or a group of similar fluorophores.

In this study, this analysis was carried out using MATLAB 18b with PARAFAC using the drEEM toolbox (ver. 0.2.0), according to the method proposed by Murphy, et al. [15]. The results were decomposed into a four-component result that explained more than 98.8% of the EEM variables.

1.3 Molecular formula assignment

Molecular formulas were assigned to all ions with signal-to-noise ratios of greater than 10 with a mass tolerance of ± 1.5 ppm using custom software. We only analyzed the data obtained in positive mode because the data obtained in negative mode were disturbed by Cl^-. Molecular formulas with their maximum numbers of atoms were defined as: 30 ^{12}C, 60 ^{1}H, 20 ^{16}O, 3 ^{14}N, 1 ^{32}S, 1 ^{13}C, 1 ^{18}O and 1 ^{34}S. Identified formulas containing isotopomers (i.e., ^{13}C, ^{18}O or ^{34}S) were not considered. Identified formulas containing isotopomers (i.e., ^{13}C, ^{18}O or ^{34}S) were not considered. For the chemical formula $C_cH_hO_oN_nS_s$, the double bond equivalence (DBE) was calculated using the following equation: DBE = $(2c+2-h+n)/2$. The details of this data processing method have previously been described [16, 17].

To identify the biomolecular class to which each molecular formula belonged, the van Krevelen diagram [18] and the modified aromaticity index (AI_{mod}) [19, 20] were used. The van Krevelen diagram is constructed using the molar ratio of hydrogen to carbon (H/C ratio) as the ordinate and the molar oxygen-to-carbon ratio (O/C ratio) as the abscissa. Major biogeochemical classes of compounds (such as lipids compounds, aliphatic/proteins, carbohydrates, etc.) have their own characteristic H/C or O/C ratios. As a result, each class of compounds plots in a specific location in the diagram. AI_{mod} is a measure of the probable aromaticity for a given molecular formula assuming that half of the oxygen atoms are doubly bound and half are present as σ bonds was calculated as: $AI_{mod} = (1 + C - 0.5O - S - 0.5[N + P + H])/(C - 0.5O - N - S - P)$. Formulas with $AI_{mod} \geqslant 0.5$ and < 0.67 are assigned as aromatics, while formulas

with AI_{mod} ⩾ 0.67 are assigned as condensed aromatics [19]. The following compound classes were defined (Table S1) based on previous studies [21, 22].

2. The description of four modeled components

Component 1 (C1) closely resembles a tyrosine-like peak B fluorophore with a excitation peak at a wavelength of 270 nm, and a emission peak at a wavelength of 315 nm. It reflects the highly biogenic degradation of amino acid containing DOM [6, 23]. Component 2 (C2) matches the microbial humic-like fluorescent peak M [24] with a excitation peak at a wavelength of < 250 nm, and a emission peak at a wavelength of 425 nm. Component 3 (C3) is composed of two excitation maxima at < 250 nm and around 300 nm and an emission maximum at 339 nm, which are similar to the components of the tryptophan-like fluorescent peak T [23]. Component 4 (C4) had excitation maxima at 290 nm and an emission maximum at 341 nm, which are also similar to the components of the tryptophan-like fluorescent peak T [23].

Table S1. Relevant common fluorophores identified in previous studies [6, 12, 23-26].

Component	Flurophore peak label	Excitation maxima nm	Emission maxima nm	Description
Tyrosine-like	B	270-275 (<240)	304-312	Amino acid, free or bound in proteins, fluorescence resembles free tyrosine, may indicate more degraded peptide material
Trytophan-like	T	270-300 (<240)	320-368	Amino acid, free or bound in proteins, fluorescence resembles free Trytophan, may indicate intact protein or less degraded peptide material
UVA Humic-like	M	<250	370-430	Oxidized, humic-like, low molecular weight correlated with aliphatic C content, associated with autochthonous production, potential photoproduct of terrestrial DOM
UVC humic-like	A	<260	448-480	High molecular weight and aromatic humic, widespread, but highest in wetlands and forested environment
UVC humic-like	C	320-360	420-460	Hight molecular weight humic, widespread, but highest in wetlands and forested environments

Table S2. Characteristics of compound classes used for categorizing FT-ICR MS molecular formulas

Compound lasses	Criterion
Lipids	$0 \leqslant O/C < 0.3, 1.5 < H/C < 2.4$
Aliphatic/proteins	$0.3 \leqslant O/C \leqslant 0.67, 1.5 < H/C < 2.4$
Carbohydrates	$0.67 < O/C < 1.2, \quad 1.5 < H/C < 2.4$
Unsaturated hydrocarbons	$0 < O/C < 0.1, 0.7 \leqslant H/C \leqslant 1.5$
Lignin/CRAM	$0.1 \leqslant O/C \leqslant 0.67, \quad 0.7 \leqslant H/C \leqslant 1.5, AI < 0.67$
Tannins	$0.67 < O/C < 1.2, 0.5 \leqslant H/C \leqslant 1.5, AI < 0.67$
Condensed aromatics	$0 < O/C \leqslant 0.67, 0.2 \leqslant H/C < 0.7, AI \geqslant 0.67$

Table S3. Average assigned molecular formula of van Krevelen chemical classes and mass content of carbon (C_m) in each average formula

Sample type	Lipids (C_m)	Aliphatic/ proteins (C_m)	Carbohydrates (C_m)	Unsaturated hydrocarbons (C_m)	Lignins/CRAM and tannins (C_m)	Condensed aromatics (C_m)
Fresh snow	$C_{21}H_{37.7}N_{0.3}O_{4.5}$ (0.689)	$C_{18.5}H_{34.3}N_{0.2}O_{7.8}$ (0.578)	$C_{12.3}H_{25}N_{0.5}O_{9.5}$ (0.445)	$C_{29.7}H_{30.3}N_{0.1}O_{1.8}$ (0.885)	$C_{22.2}H_{28.7}N_{0.2}O_{6.3}$ (0.668)	$C_{27.6}H_{15.3}N_{0.1}O_{3.1}$ (0.833)
Fine firn	$C_{21.2}H_{38.3}N_{0.3}O_{4.2}$ (0.699)	$C_{18.1}H_{34}N_{0.2}O_{7.6}$ (0.578)	$C_{11}H_{21.8}N_{0.4}O_{8.7}$ (0.440)	$C_{28.7}H_{20.2}N_{0.1}O_{1.9}$ (0.869)	$C_{19.8}H_{25.9}N_{0.2}O_{5.4}$ (0.674)	$C_{28.1}H_{15.4}N_{0.04}O_{3.1}$ (0.837)
Coarse firn	$C_{21.1}H_{37.7}N_{0.3}O_{4.4}$ (0.693)	$C_{19.8}H_{36.3}N_{0.2}O_{8.1}$ (0.585)	$C_{11.2}H_{22.1}N_{0.4}O_{8.8}$ (0.444)	$C_{32}H_{32.9}N_{0.2}O_{1.9}$ (0.853)	$C_{20.2}H_{26.7}N_{0.3}O_{5.9}$ (0.659)	$C_{30.1}H_{18.7}N_{0.1}O_{2.3}$ (0.864)
Granular ice	$C_{21}H_{37.8}N_{0.3}O_{4.4}$ (0.692)	$C_{19.8}H_{36.2}N_{0.3}O_{8.2}$ (0.581)	$C_{11.9}H_{24.4}N_{0.7}O_{9.1}$ (0.443)	$C_{26.8}H_{28.2}N_{0.1}O_{1.7}$ (0.850)	$C_{20.8}H_{27.5}N_{0.3}O_{6.3}$ (0.653)	$C_{28.2}H_{15.4}N_{0}O_{2.7}$ (0.852)

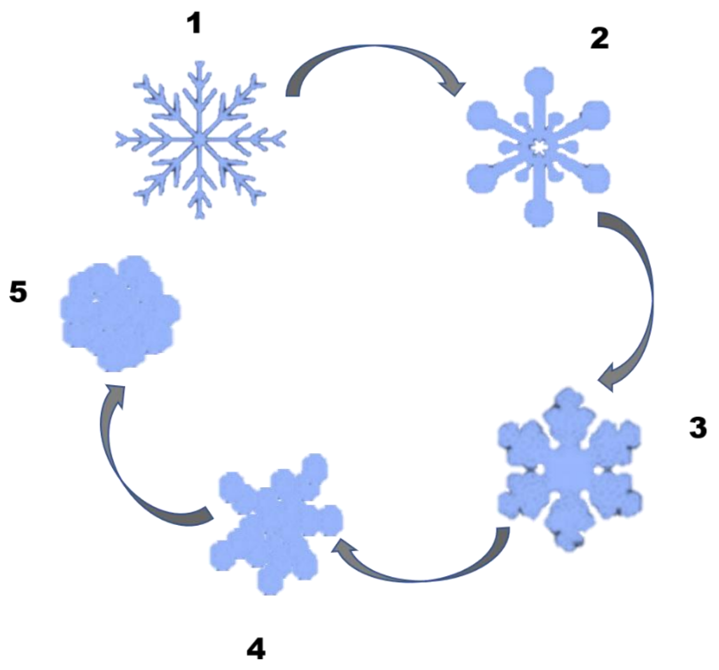

Figure S1. The metamorphism (physical evolution) of snow and corresponding snow/ice category during snowmelt: fresh snow (stage 1), fine firn (stage 2), coarse firn (stage 3), granular ice (stage 4) and glacier ice (stage 5) [27]. The figure is generated in Microsoft PowerPoint 2019 by the first author of Lin Feng.

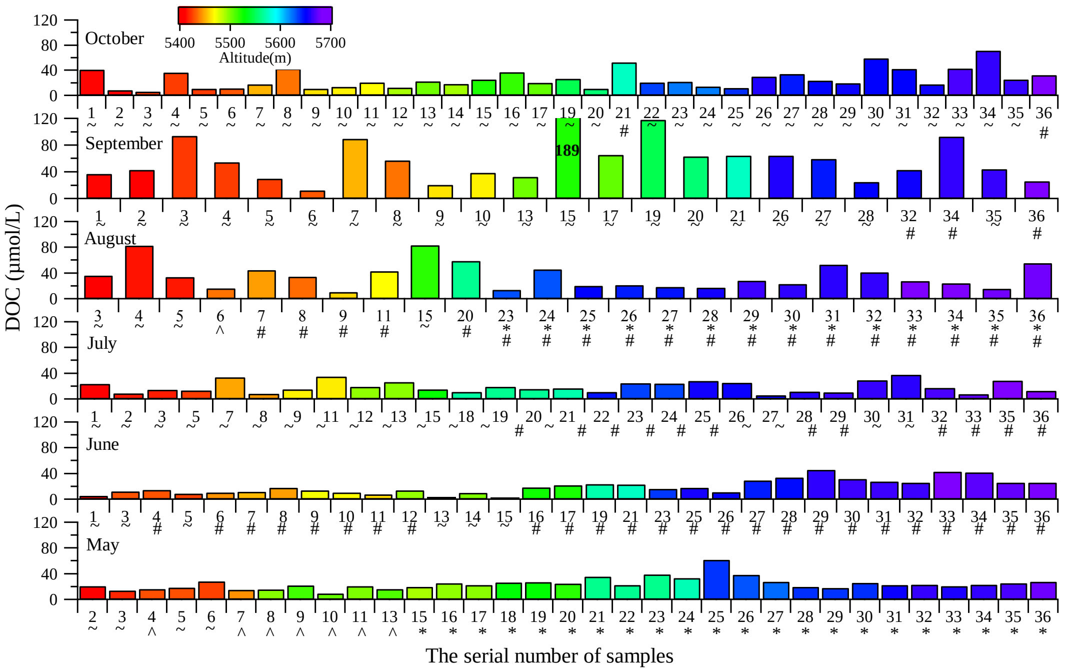

Figure S2. DOC concentrations in each snow/ice sample in different months colored according to sampling altitude. The number of the samples obtained in each month was normally 36, but one or two samples were omitted due to running out of for other measurement. The symbol under the number of each sample denotes its physical properties (* fresh snow, ^ fine firn, # coarse firn, ~ granular ice).

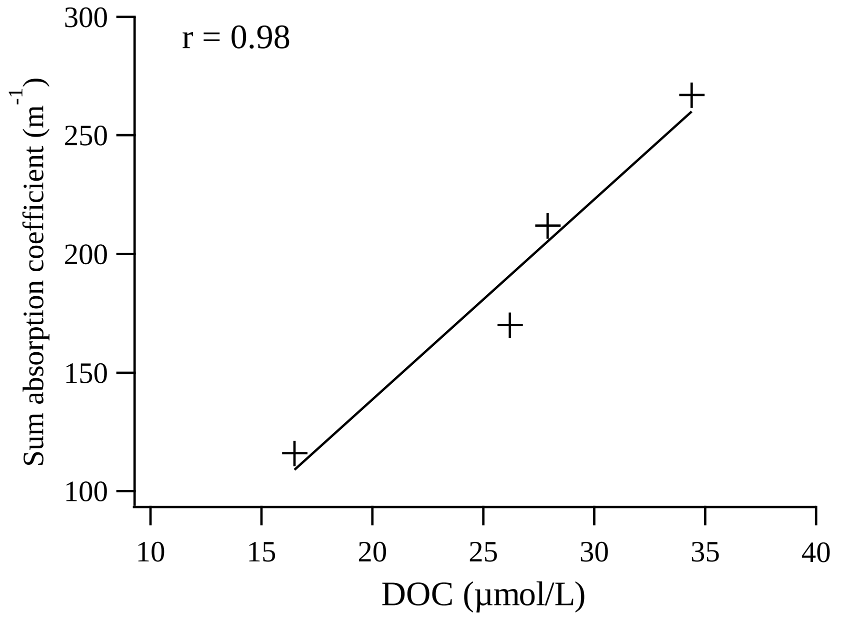

Figure S3. Scatter plots of sum of absorption coefficient (between 220-450 nm) versus dissolved organic carbon (DOC) in each fresh snow.

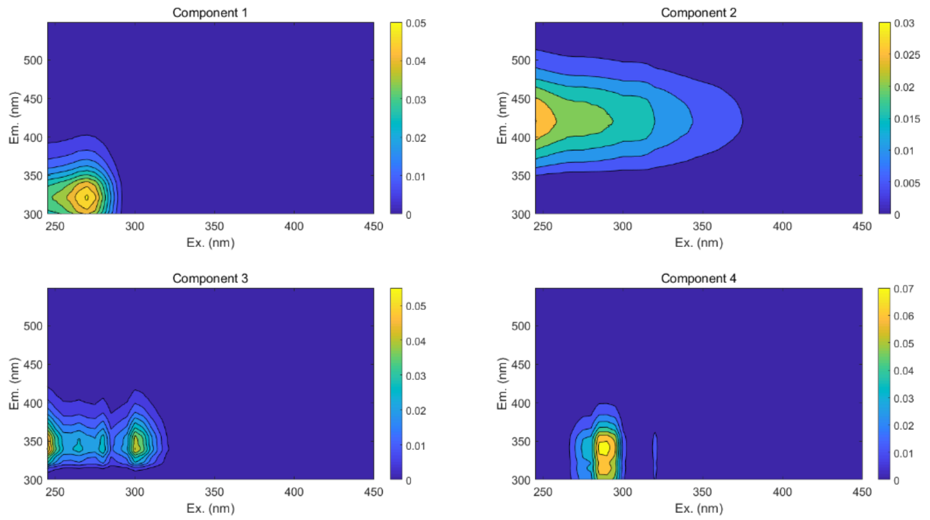

Figure S4. Excitation-emission matrices (EEMs) for the four-component PARAFAC model.

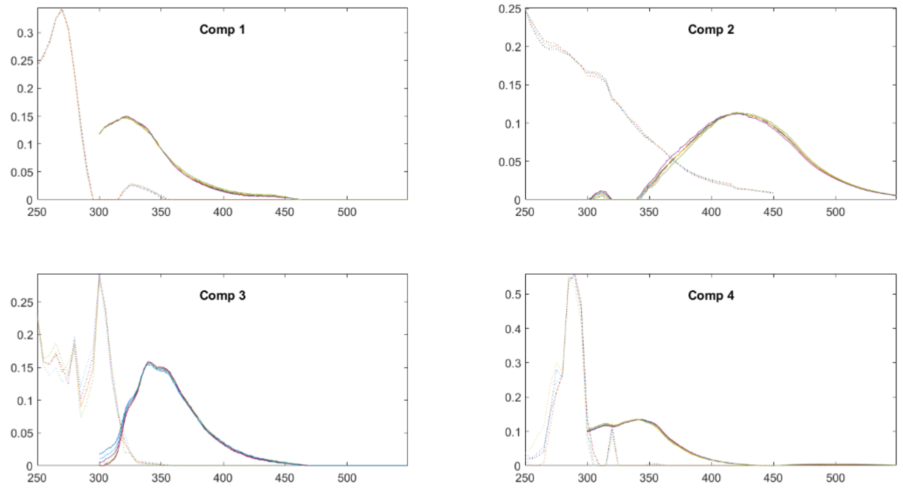

Figure S5. Results of the split-half analysis of the 4-Component PARAFAC model using 4 splits.

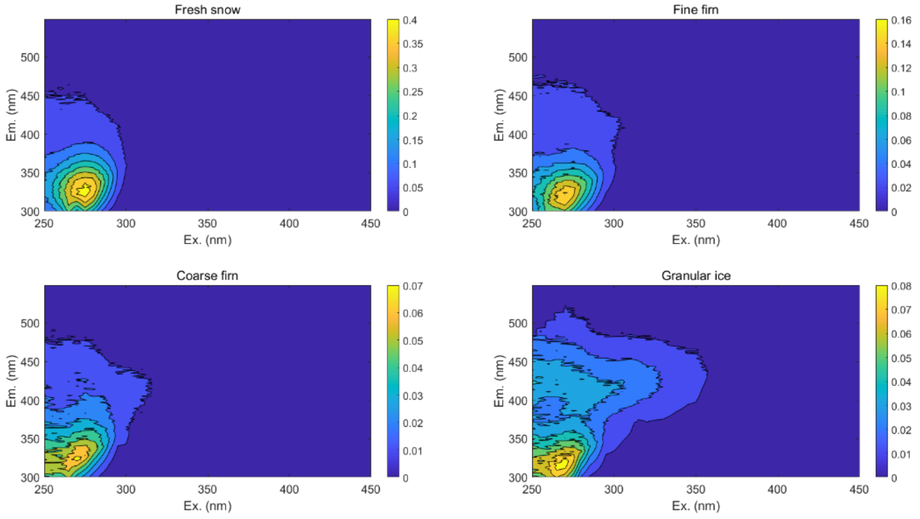

Figure S6. The typical EEM spectra, e.g., for fresh snow, fine firn, coarse firn, and granular ice

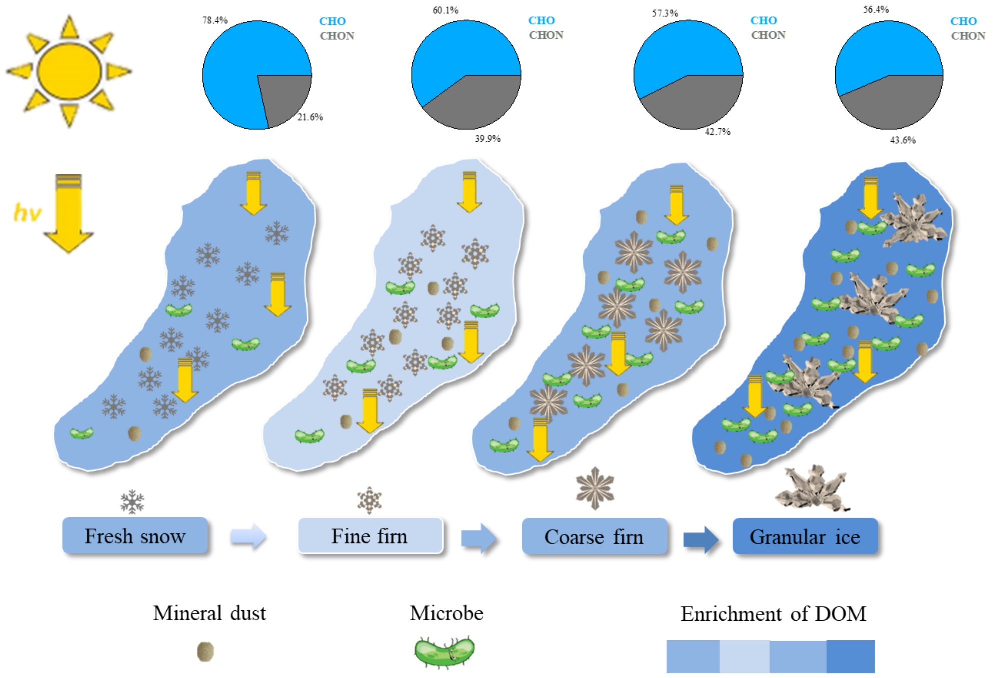

Figure S7. The schematic plot for the evolution of snow physics, microbial activity, and DOM content during snowmelt in the glacier.

Reference

1. Helms, J. R. *et al.* Absorption spectral slopes and slope ratios as indicators of molecular weight, source, and photobleaching of chromophoric dissolved organic matter. *Limnol. Oceanogr.* **53**, 955–969 (2008).

2. Beine, H. *et al.* Soluble, light–absorbing species in snow at Barrow, Alaska. *J. Geophys. Res.: Atmos.* **116**, D14 (2011).

3. Weishaar, J. L. *et al.* Evaluation of specific ultraviolet absorbance as an indicator of the chemical composition and reactivity of dissolved organic carbon. *Environ. Sci. Technol.* **37**, 4702–4708 (2003).

4. Cory, R. M. & McKnight, D. M. Fluorescence Spectroscopy Reveals Ubiquitous Presence of Oxidized and Reduced Quinones in Dissolved Organic Matter. *Environ. Sci. Technol.* **39**, 8142–8149 (2005).

5. Birdwell, J. E. & Engel, A. S. Characterization of dissolved organic matter in cave and spring waters using UV-Vis absorbance and fluorescence spectroscopy. *Organic Geochemistry* **41**, 270-280 (2010).

6. Fellman, J. B., Hood, E. & Spencer, R. G. M. Fluorescence spectroscopy opens new windows into dissolved organic matter dynamics in freshwater ecosystems: a review. *Limnology & Oceanography* **55**, 2452-2462 (2010).

7. Zhou, Y. *et al.* Lake Taihu, a large, shallow and eutrophic aquatic ecosystem in China serves as a sink for chromophoric dissolved organic matter. *Journal of Great Lakes Research* **41**, 597-606 (2015).

8. Bahram, M., Bro, R., Stedmon, C. & Afkhami, A. Handling of Rayleigh and Raman scatter for PARAFAC modeling of fluorescence data using interpolation. *Journal of Chemometrics: A Journal of the Chemometrics Society* **20**, 99-105 (2006).

9. Hur, J., Lee, D.-H. & Shin, H.-S. Comparison of the structural, spectroscopic and phenanthrene binding characteristics of humic acids from soils and lake sediments. *Organic Geochemistry* **40**, 1091-1099 (2009).

10. Lawaetz, A. J. & Stedmon, C. A. Fluorescence intensity calibration using the Raman scatter peak of water. *Applied Spectroscopy* **63**, 936 (2009).

11. Barker, J. D., Sharp, M. J. & Turner, R. J. Using synchronous fluorescence spectroscopy and principal components analysis to monitor dissolved organic

matter dynamics in a glacier system. *Hydrological Processes* **23**, 1487-1500 (2009).

12. Dubnick, A. *et al.* Characterization of dissolved organic matter (DOM) from glacial environments using total fluorescence spectroscopy and parallel factor analysis. *Annals of Glaciology* **51**, 111-122 (2010).
13. D'Andrilli, J., Foreman, C. M., Sigl, M., Priscu, J. C. & Mcconnell, J. R. A 21 000-year record of fluorescent organic matter markers in the WAIS Divide ice core. *Climate of the Past* **13**, 1–15 (2017).
14. Barker, J. D., Dubnick, A., Lyons, W. B. & Chin, Y. P. Changes in Dissolved Organic Matter (DOM) Fluorescence in Proglacial Antarctic Streams. *Arctic Antarctic & Alpine Research* **45**, 305-317 (2013).
15. Murphy, K. R., Stedmon, C. A., Graeber, D. & Bro, R. Fluorescence spectroscopy and multi-way techniques. PARAFAC. *Anal. Methods* **5**, 6557–6566 (2013).
16. Jiang, B. *et al.* Polycyclic aromatic hydrocarbons (PAHs) in ambient aerosols from Beijing: characterization of low volatile PAHs by positive-ion atmospheric pressure photoionization (APPI) coupled with Fourier transform ion cyclotron resonance. *Environmental Science & Technology* **48**, 4716 (2014).
17. Quan, S. *et al.* Characterization of Middle-Temperature Gasification Coal Tar. Part 3: Molecular Composition of Acidic Compounds. *Energy Fuels* **27**, 108–117 (2013).
18. Kim, S., And, R. W. K. & Hatcher, P. G. Graphical Method for Analysis of Ultrahigh-Resolution Broadband Mass Spectra of Natural Organic Matter, the Van Krevelen Diagram. *Anal. Chem.* **75**, 5336–5344 (2003).
19. Koch, B. & Dittmar, T. From mass to structure: an aromaticity index for high‐resolution mass data of natural organic matter. *Rapid Commun. Mass Spectrom.* **20**, 926‐932 (2006).
20. Koch, B. & Dittmar, T. From mass to structure: an aromaticity index for high‐resolution mass data of natural organic matter. *Rapid Commun. Mass Spectrom.* **30**, 250‐250 (2016).
21. Grannas, A. M., Hockaday, W. C., Hatcher, P. G., Thompson, L. G. & Ellen, M. T. New revelations on the nature of organic matter in ice cores. *Journal of Geophysical Research Atmospheres* **111**, 613-666 (2006).

22. Hockaday, W. C., Purcell, J. M., Marshall, A. G., Baldock, J. A. & Hatcher, P. G. Electrospray and photoionization mass spectrometry for the characterization of organic matter in natural waters: a qualitative assessment. *Limnology & Oceanography Methods* **7**, 81-95 (2009).

23. Coble, P. G., Del Castillo, C. E. & Avril, B. Distribution and optical properties of CDOM in the Arabian Sea during the 1995 Southwest Monsoon. *Deep Sea Research Part II Topical Studies in Oceanography* **45**, 2195-2223 (1998).

24. And, R. M. C. & Mcknight, D. M. Fluorescence Spectroscopy Reveals Ubiquitous Presence of Oxidized and Reduced Quinones in Dissolved Organic Matter. *Environmental Science & Technology* **39**, 8142-8149 (2005).

25. Coble, P. G., Green, S. A., Blough, N. V. & Gagosian, R. B. Characterization of dissolved organic matter in the Black Sea by fluorescence spectroscopy. *Nature* **348**, 432 (1990).

26. Parlanti, E., Wörz, K., Geoffroy, L. & Lamotte, M. Dissolved organic matter fluorescence spectroscopy as a tool to estimate biological activity in a coastal zone submitted to anthropogenic inputs. *Organic Geochemistry* **31**, 1765-1781 (2000).

27. Hock, R. Glacier melt: a review of processes and their modelling. *Progress in physical geography* **29**, 362–391 (2005).
